# Supplementary material for: Endothelin-1 is associated with mortality that can be attenuated with high intensity statin therapy in patients with stable coronary artery disease
Source: Commun Med (Lond). 2023 Jun 22;3:87. doi: 10.1038/s43856-023-00322-9 (PMC10287654; doi:10.1038/s43856-023-00322-9)
Supplement: Supplementary file 1 — Description of Additional Supplementary Files [file 43856_2023_322_MOESM1_ESM.pdf]

Description of Additional Supplementary File

File Name: Supplementary Data 1

Description:

Title:
Unadjusted and multivariate-adjusted association of ET-1 level and clinical outcomes
Legend:
BMI, body mass index; sysBP, systolic blood pressure; diaBP, diastolic blood pressure; BNP, B-type natriuretic peptide ; LVEF, left ventricular ejection fraction ; ET-1, endothelin-1; GHbA1C %, percentage of glycated hemoglobin A1c ; LDL, low-density lipoprotein; HDL, high-density lipoprotein; NYHA, New York Heart Association classification; MI, myocardial infarction; CABG, coronary artery bypass surgery; PCI, percutaneous coronary intervention; T2D, type 2 diabetes; CV death, cardiovascular death; non-CV death, non-cardiovascular death; SCD, sudden cardiac death. The bold format was used to highlight ET-1.

File Name: Supplementary Data 2

Description:

Title:
Statins and risk for all-cause mortality and CV-death in high ET-1 group
Legend:
BMI, body mass index; sysBP, systolic blood pressure; diaBP, diastolic blood pressure; BNP, B-type natriuretic peptide ; LVEF, left ventricular ejection fraction ; ET-1, endothelin-1; GHbA1C %, percentage of glycated hemoglobin A1c ; LDL, low-density lipoprotein; HDL, high-density lipoprotein; NYHA, New York Heart Association classification; MI, myocardial infarction; CABG, coronary artery bypass surgery; PCI, percutaneous coronary intervention; T2D, type 2 diabetes; CV death, cardiovascular death. The bold format was used to highlight groups of statins.

File Name: Supplementary Data 3

Description:

Title:
Statins and risk for all-cause mortality and CV-death in high ET-1 group.
Legend:
NYHA, New York Heart Association Classification; ARBs, Angiotensin II receptor blocker; ACEIs, Angiotensin-Converting Enzyme Inhibitors ; β-blockers, β-adrenergic blockers; CCBs, calcium channel blockers; T2D, type 2 diabetes; CV death, cardiovascular death. The bold format was used to highlight groups of statins.
