# Supplementary material for: Endothelin-1 is associated with mortality that can be attenuated with high intensity statin therapy in patients with stable coronary artery disease
Source: Commun Med (Lond). 2023 Jun 22;3:87. doi: 10.1038/s43856-023-00322-9 (PMC10287654; doi:10.1038/s43856-023-00322-9)
Supplement: Supplementary file 2 — Supplementary Information [file 43856_2023_322_MOESM2_ESM.pdf]

Supplementary Information

Endothelin-1 is associated with mortality that can be attenuated with statin therapy in patients with stable coronary artery disease

Ruizhu Lin[1], Juhani Junttila1[1,2,3], Jarkko Piuhola[1], E. Samuli Lepojärvi[1], Johanna Magga[1,3], Antti M. Kiviniemi[1], Juha Perkiömäki[1], Heikki Huikuri[1], Olavi Ukkola[1], Mikko Tulppo[1], Risto Kerkelä[1,2,3]

1 Research Unit of Biomedicine and Internal Medicine, University of Oulu, Oulu, Finland

2 Medical Research Centre Oulu, Oulu University Hospital and University of Oulu, Oulu, Finland

3 Biocenter Oulu, University of Oulu, Oulu, Finland.

Figure S1

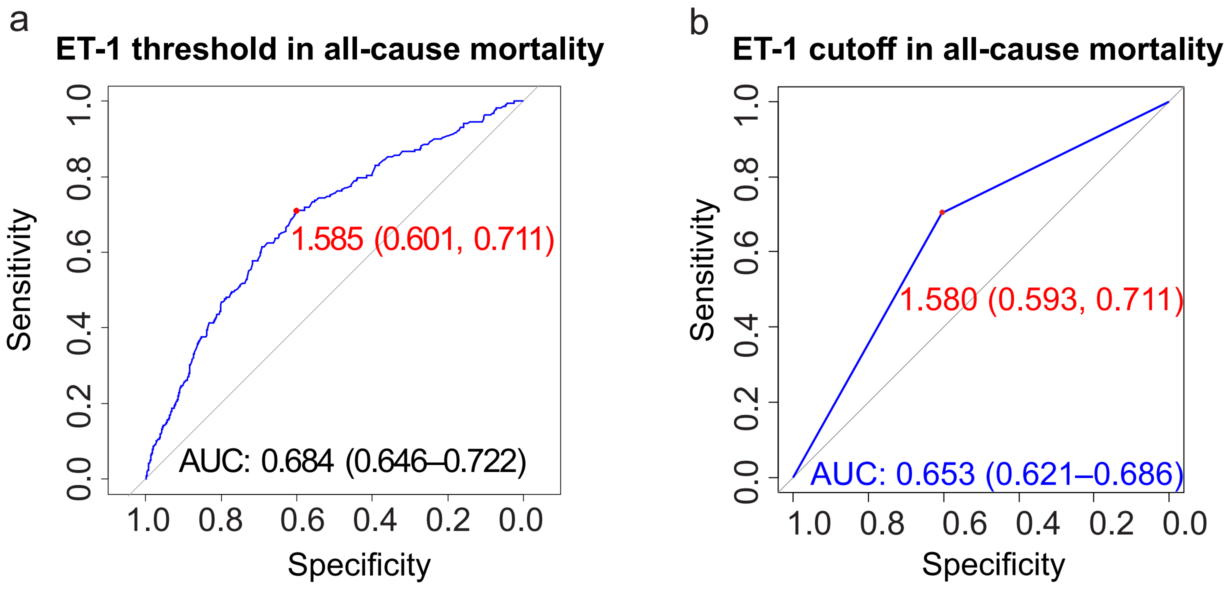

Figure S1. ROC curves for ET-1 to discriminate all-cause mortality.

Receiver-operating characteristic (ROC) curve analysis was conducted to obtain the cutoff value of ET-1 for optimal sensitivity and specificity. (a) ET-1 was used as continuous variable to predict all-cause mortality, and the estimated optimal thershold of concentration was 1.585 pg/ml (AUC=0.684 [95% CI, 0.646–0.722], specificity=0.601, sensitivity=0.711). (b) ET-1 was used as cutoff of 1.580 pg/ml (AUC=0.653 [95% CI, 0.621–0.686], specificity=0.593, sensitivity=0.711). (a-b) n=1945 biologically independent samples. ET-1, endothelin-1; AUC, area under the curve

Figure S2

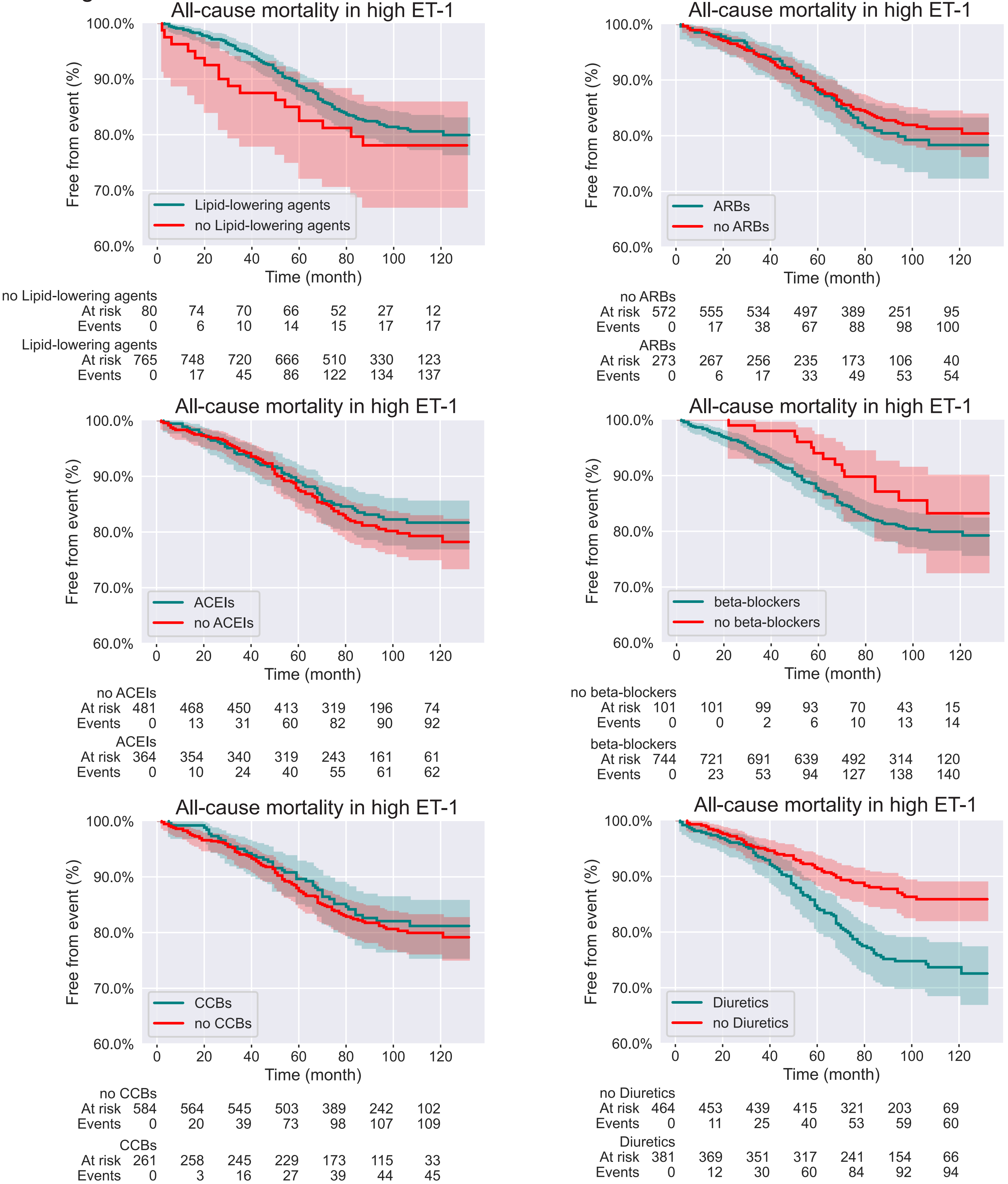

Figure S2. Kaplan-Meier curves for primary endpoint in high ET-1 group upon various medication. Shown are rate of freedom from all-cause mortality over follow-up time. Chi-square and p value were derived from log-rank test between low and high ET-1 groups. Shadows indicated confident interval of estimates. ARBs, Angiotensin II receptor blocker; ACEIs, Angiotensin-Converting Enzyme Inhibitors ; beta-blockers, beta-adrenergic blockers; CCBs, calcium channel blockers; ET-1, endothelin-1.

Figure S3

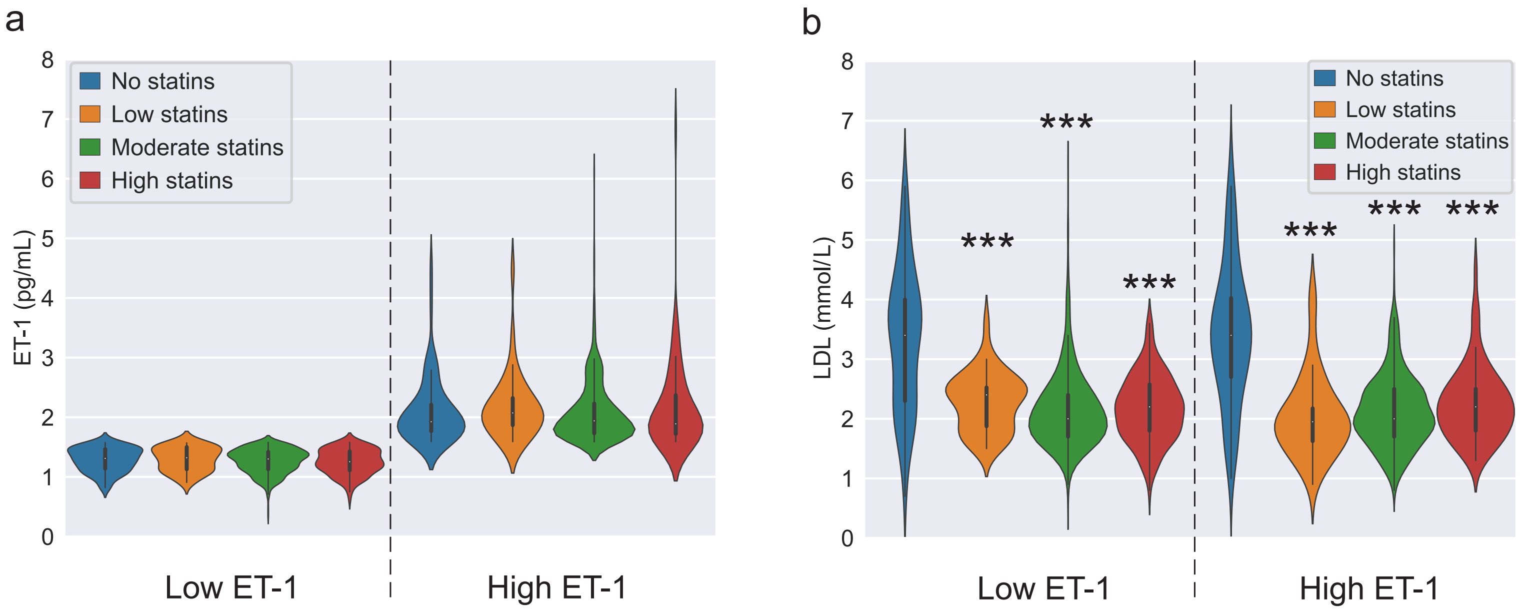

Figure S3. Analysis for circulating ET-1 and LDL concentration in subgroups.
Shown are violin plots of circulating ET-1 (a) and LDL (b) levels according to ET-1 level and statin use. Inside is a miniature of boxplot. In low ET-1 group, No statins: n=87, Low statins: n=36, Moderate statins: n=654, High statins: n=138; In high ET-1 group, No statins: n=80, Low statins: n=38, Moderate statins: n=462, High statins: n=69 biologically independent samples. ***p<0.001 vs No statins group. Wilcoxon rank-sum test was used for statistical testing due to non-normatlity. ET-1,endothelin-1; LDL, Low-density lipoprotein. The vertical dash lines were used to seperate low and high ET-1 groups.

Figure S4

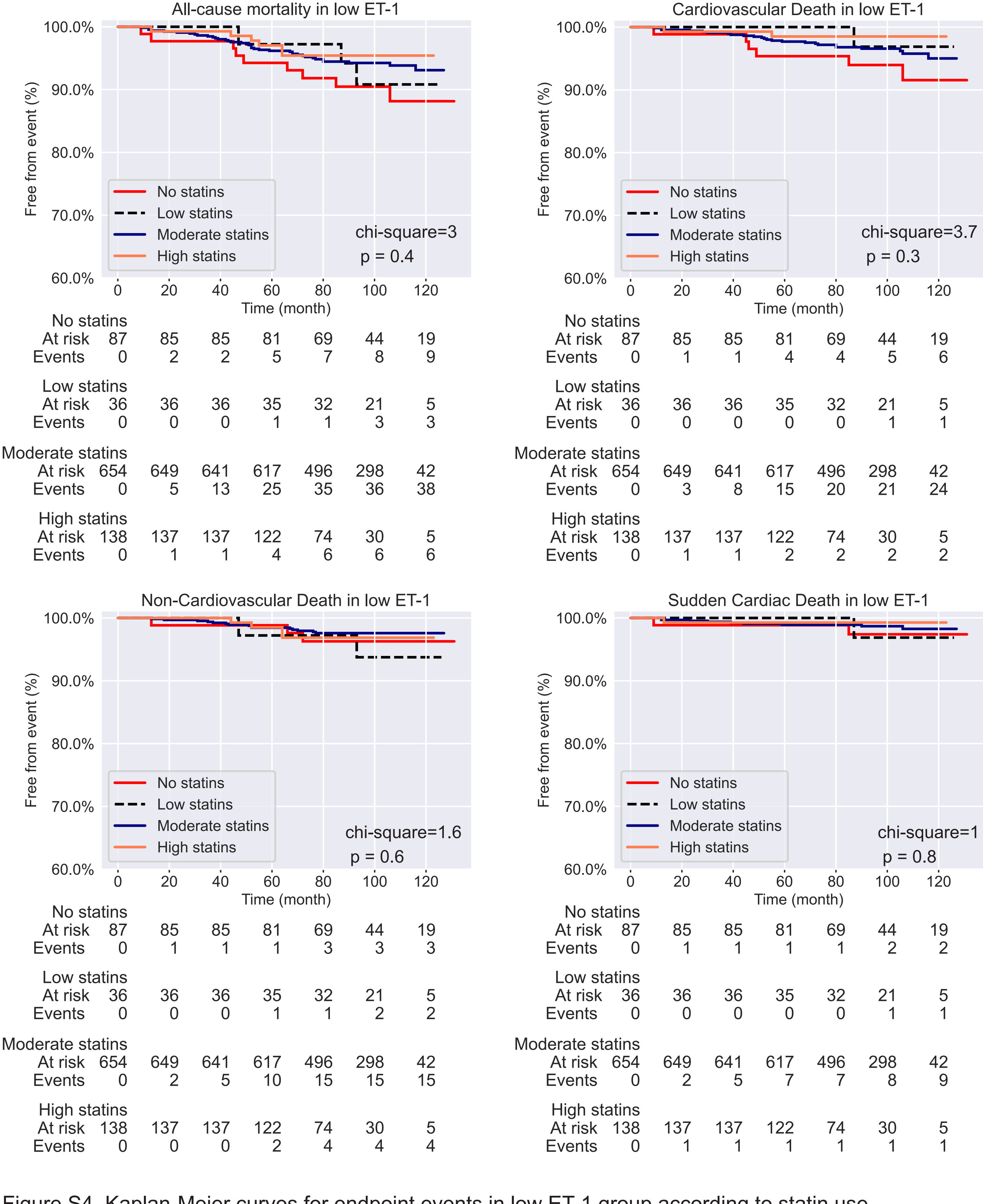

Figure S4. Kaplan-Meier curves for endpoint events in low ET-1 group according to statin use. Shown are rate of freedom from all-cause mortality, cardiovascular death, non-cardiovascular death and sudden cardiac death over follow-up time. Chi-square and p value were derived from log-rank test between statin groups. ET-1, endothelin-1.

Table S1. Baseline characteristics of patients in ET-1 quartiles

	Overall	Q1	Q2	Q3	Q4	p
N	1945	494	479	486	486	
Age (year)	67 [61, 73]	64 [59, 70]	67 [61, 72]	69 [63, 74]	70 [64, 75]	<0.0001
T2D (%)	833 (42.8)	106 (21.5)	175 (36.5)	243 (50.0)	309 (63.6)	<0.0001
Sex (%) (female)	618 (31.8)	125 (25.3)	143 (29.9)	160 (32.9)	190 (39.1)	<0.0001
LVEF (%)	65.50 [59.95, 70.10]	65.40 [60.35, 70.30]	66.00 [60.82, 69.88]	65.10 [59.08, 69.60]	65.20 [58.10, 71.00]	0.3552
BMI (kg/m^2)	28.00 [25.00, 31.00]	27.00 [25.00, 29.00]	28.00 [25.00, 31.00]	28.00 [25.00, 31.00]	29.00 [26.00, 33.00]	<0.0001
sysBP (mm Hg)	145 [129, 162]	138.50 [123.25, 154]	146 [129, 164]	149 [134, 163]	147 [131, 163.75]	<0.0001
diaBP (mm Hg)	77 [70, 84]	76 [69, 83]	77 [71, 84]	78 [70, 85]	77 [70, 85]	0.0090
BNP (pg/mL)	50.00 [25.00, 93.00]	38.00 [20.00, 73.00]	49.00 [24.00, 86.00]	51.00 [26.00, 99.50]	62.50 [31.25, 139.50]	<0.0001
Creatinine clearance (mL/min)	87.92 [70.38, 112.39]	90.84 [74.39, 112.99]	89.12 [73.62, 114.52]	83.63 [66.77, 109.82]	86.41 [66.45, 109.72]	0.0007
Insulin (mIU / L)	11.80 [7.30, 18.70]	9.90 [6.60, 14.30]	11.40 [7.20, 17.85]	12.40 [7.82, 19.42]	14.40 [8.40, 22.50]	<0.0001
GHbA1C (%)	6.10 [5.70, 6.60]	5.90 [5.60, 6.30]	6.00 [5.70, 6.40]	6.20 [5.80, 6.80]	6.50 [5.90, 7.20]	<0.0001
Total cholesterol (mmol/L)	3.80 [3.40, 4.40]	3.80 [3.40, 4.40]	3.80 [3.40, 4.30]	3.90 [3.40, 4.50]	3.80 [3.40, 4.40]	0.2918
HDL (mmol/L)	1.23 [1.03, 1.44]	1.25 [1.05, 1.50]	1.23 [1.05, 1.44]	1.21 [1.03, 1.44]	1.20 [1.01, 1.40]	0.0187
LDL (mmol/L)	2.10 [1.80, 2.60]	2.10 [1.80, 2.60]	2.10 [1.80, 2.60]	2.20 [1.80, 2.60]	2.10 [1.80, 2.60]	0.3977
Triglyceride (mmol/L)	1.21 [0.90, 1.67]	1.08 [0.82, 1.42]	1.18 [0.88, 1.62]	1.28 [0.96, 1.76]	1.34 [1.01, 1.79]	<0.0001
Resting heart rate (beats/min)	59 [53, 66]	58 [52, 63]	58 [53, 64]	60 [54, 67]	62 [55, 72]	<0.0001
NYHA (%)						
NYHA Class I	853 (44.3)	142 (29.0)	191 (40.6)	243 (50.2)	277 (57.5)	
NYHA Class II	69 (3.6)	15 (3.1)	15 (3.2)	12 (2.4)	27 (5.6)	
Medication (%)						
ARBs	558 (28.7)	104 (21.1)	148 (31.0)	135 (27.8)	171 (35.3)	<0.0001
ACEIs	783 (40.3)	179 (36.2)	183 (38.3)	224 (46.1)	197 (40.6)	0.0118
CCBs	474 (24.4)	69 (14.0)	106 (22.2)	139 (28.6)	160 (32.9)	<0.0001
Diuretics	666 (34.3)	90 (18.2)	148 (31.0)	178 (36.6)	250 (51.4)	<0.0001
β-blockers	1704 (87.7)	424 (85.8)	421 (88.1)	438 (90.1)	421 (86.6)	0.1862
Lipid-lowering therapy	1777 (91.4)	458 (92.7)	439 (91.8)	439 (90.3)	441 (90.7)	0.5358
History (%)						
Prior MI	929 (47.8)	247 (50.0)	216 (45.2)	237 (48.8)	229 (47.1)	0.4687
Prior PCI or CABG	1544 (79.6)	394 (80.1)	399 (83.5)	368 (76.2)	383 (78.8)	0.0432
Smoking	167 (8.6)	26 (5.3)	34 (7.1)	44 (9.1)	63 (13.0)	0.0001
Death (%)						
All-cause mortality	218 (11.2)	26 (5.3)	30 (6.3)	57 (11.7)	105 (21.6)	<0.0001
Cardiovascular Death	116 (6.0)	13 (2.6)	17 (3.5)	33 (6.8)	53 (10.9)	<0.0001
non-Cardiovascular Death	102 (5.2)	13 (2.6)	14 (2.9)	23 (4.7)	52 (10.7)	<0.0001
Sudden Cardiac Death	50 (2.6)	6 (1.2)	7 (1.5)	16 (3.3)	21 (4.3)	<0.0001

Values were expressed as mean ± SD, median [IQR], or counts (%) as appropriate. T2D, type 2 diabetes; LVEF, left ventricular ejection fraction; BMI, body mass index; sysBP, systolic blood pressure; diaBP, diastolic blood pressure; BNP, B-type natriuretic peptide ; %GHbA1C, percentage of glycated hemoglobin A1c; HDL, high-density lipoprotein; LDL, low-density lipoprotein; NYHA, New York Heart Association Classification; ARBs, Angiotensin II receptor blocker; ACEIs, Angiotensin-Converting Enzyme Inhibitors ; β-blockers, β-adrenergic blockers; CCBs, calcium channel blockers; MI, myocardial infarction; CABG, coronary artery bypass surgery; PCI, percutaneous coronary intervention. ET-1, endothelin-1.

Table S2. Baseline characteristics of patients with various statin dosage in high or low ET-1 group

	Overall	Low ET-1				high ET-1				p (low vs high ET-1)
		no statin	low statins	moderate statins	high statins	no statin	low statins	moderate statins	high statins	
N	1564	87	36	654	138	80	38	462	69	
Age (year)	68 [62, 74]	66 [60, 74]	69.50 [65.75, 75.25]	66 [61, 72]	63 [54.25, 67]	70.50 [64, 75]	74 [69, 76.75]	70 [64, 76]	67 [61, 74]	<0.0001
T2D (%)	605 (38.7)	26 (29.9)	14 (38.9)	175 (26.8)	45 (32.6)	49 (61.3)	19 (50.0)	236 (51.1)	41 (59.4)	<0.0001
Sex (%) (female)	487 (31.1)	28 (32.2)	14 (38.9)	172 (26.3)	36 (26.1)	30 (37.5)	17 (44.7)	162 (35.1)	28 (40.6)	0.0001
LVEF (%)	65.60 [60.02, 70.07]	65.60 [61.40, 70.53]	65.05 [60.22, 70.90]	65.88 [60.90, 70.11]	64.80 [60.30, 67.80]	64.25 [57.48, 71.10]	65.90 [58.50, 71.22]	65.70 [59.20, 70.00]	64.40 [58.00, 69.50]	0.4235
BMI (kg/m^2)	27.00 [25.00, 30.00]	27.00 [24.74, 30.50]	26.00 [24.00, 29.00]	27.00 [25.00, 29.00]	27.00 [25.00, 30.00]	28.00 [24.00, 32.00]	28.20 [24.25, 30.75]	28.00 [26.00, 32.00]	29.00 [26.00, 32.00]	<0.0001
sysBP (mm Hg)	145 [129, 162]	145 [125, 164]	152.50 [132, 164]	144 [128, 159]	135 [123, 153]	150.50 [134.75, 168.75]	135 [127.25, 160]	148 [133, 165]	142 [128, 153]	0.0001
diaBP (mm Hg)	76 [70, 84]	75 [69, 84]	78 [73.75, 85]	76 [70, 83]	77 [70, 83]	77 [71.75, 86]	72.50 [68, 80.75]	77 [70, 85.75]	74 [69, 81]	0.2833
BNP (pg/mL)	50.50 [25.00, 97.00]	39.00 [18.50, 86.00]	43.50 [21.25, 86.75]	48.50 [25.00, 83.75]	37.00 [17.25, 76.25]	60.50 [33.50, 139.75]	84.50 [39.25, 143.25]	58.00 [29.00, 123.00]	59.00 [29.00, 132.00]	<0.0001
Creatinine clearance (mL/min)	86.41 [69.40, 109.93]	92.25 [75.14, 111.39]	86.42 [72.97, 100.82]	86.10 [70.17, 109.83]	101.87 [82.55, 126.73]	86.12 [65.96, 109.63]	77.53 [65.84, 96.30]	81.81 [63.72, 105.68]	95.05 [68.95, 119.03]	0.0001
Insulin (mlU / L)	11.30 [7.20, 18.00]	9.20 [5.55, 14.90]	10.95 [6.00, 14.82]	9.90 [6.90, 15.60]	12.30 [7.40, 19.55]	12.90 [7.30, 23.85]	11.65 [6.35, 21.23]	12.75 [7.80, 20.28]	14.20 [8.95, 23.12]	<0.0001
GHbA1C (%)	6.00 [5.70, 6.50]	5.90 [5.60, 6.40]	6.05 [5.80, 6.43]	5.90 [5.60, 6.30]	5.95 [5.62, 6.30]	6.35 [5.97, 6.98]	6.20 [5.70, 6.97]	6.20 [5.80, 6.90]	6.10 [5.90, 6.60]	<0.0001
Total cholesterol (mmol/L)	3.80 [3.40, 4.40]	5.10 [4.20, 5.95]	3.90 [3.70, 4.20]	3.70 [3.30, 4.20]	3.90 [3.40, 4.30]	5.20 [4.50, 5.73]	3.65 [3.35, 4.27]	3.70 [3.30, 4.20]	3.90 [3.50, 4.40]	**0.4865**
HDL (mmol/L)	1.24 [1.04, 1.46]	1.25 [1.11, 1.54]	1.25 [1.05, 1.45]	1.25 [1.06, 1.47]	1.19 [1.00, 1.43]	1.25 [1.02, 1.45]	1.27 [1.12, 1.50]	1.23 [1.02, 1.43]	1.11 [0.97, 1.35]	**0.0472**
LDL (mmol/L)	2.10 [1.80, 2.60]	3.40 [2.30, 4.00]	2.40 [1.87, 2.52]	2.00 [1.70, 2.40]	2.20 [1.80, 2.58]	3.40 [2.70, 4.03]	1.95 [1.63, 2.18]	2.00 [1.70, 2.50]	2.20 [1.80, 2.50]	**0.7366**
Triglyceride (mmol/L)	1.18 [0.89, 1.62]	1.29 [0.92, 1.64]	1.08 [0.81, 1.28]	1.10 [0.83, 1.54]	1.17 [0.91, 1.57]	1.54 [1.08, 2.05]	1.21 [0.89, 1.62]	1.25 [0.96, 1.68]	1.36 [1.03, 2.01]	**<0.0001**
Resting heart rate (beats/min)	59 [53, 66]	58.50 [52, 65]	56 [50, 62.50]	57 [52, 64]	58 [53, 64]	64 [58, 76]	62 [56, 67]	61 [54, 68]	60 [54.50, 68]	<0.0001
NYHA (%)										
NYHA Class I	689 (44.5)	31 (36.0)	15 (41.7)	220 (34.1)	55 (40.1)	45 (57.0)	21 (55.3)	259 (56.4)	43 (62.3)	<0.0001
NYHA Class II	52 (3.3)	2 (2.3)	0 (0.0)	19 (3)	4 (2.9)	8 (10.2)	4 (10.5)	13 (2.9)	2 (2.9)	
Medication (%)										
ARBs	438 (28.0)	20 (23.0)	12 (33.3)	162 (24.8)	39 (28.3)	20 (25.0)	14 (36.8)	146 (31.7)	25 (36.2)	0.0092
ACEIs	631 (40.4)	31 (35.6)	17 (47.2)	253 (38.7)	55 (39.9)	32 (40.0)	13 (34.2)	199 (43.2)	31 (44.9)	0.1772
CCBs	373 (23.8)	17 (19.5)	16 (44.4)	122 (18.7)	20 (14.5)	18 (22.5)	19 (50.0)	135 (29.2)	26 (37.7)	<0.0001
Diuretics	515 (32.9)	23 (26.4)	13 (36.1)	159 (24.3)	33 (23.9)	27 (33.8)	24 (63.2)	205 (44.4)	31 (44.9)	<0.0001
β-blockers	1360 (87.0)	68 (78.2)	33 (91.7)	575 (87.9)	119 (86.2)	64 (80.0)	33 (86.8)	406 (87.9)	62 (89.9)	0.9815
Lipid-lowering therapy	1397 (89.3)	0 (0.0)	36 (100.0)	654 (100.0)	138 (100.0)	0 (0.0)	38 (100.0)	462 (100.0)	69 (100.0)	0.0900
History (%)										
Prior MI	754 (48.2)	29 (33.3)	14 (38.9)	315 (48.2)	85 (61.6)	30 (37.5)	16 (42.1)	226 (48.9)	39 (56.5)	0.8872
Prior PCI or CABG	1236 (79.2)	49 (57.6)	25 (69.4)	528 (81.0)	125 (90.6)	50 (62.5)	31 (81.6)	365 (79.0)	63 (91.3)	0.5511
Smoking	131 (8.4)	8 (9.2)	2 (5.6)	34 (5.2)	10 (7.2)	13 (16.5)	7 (18.4)	49 (10.6)	8 (11.6)	<0.0001
Death (%)										
All-cause mortality	165 (10.5)	9 (10.3)	3 (8.3)	38 (5.8)	6 (4.3)	17 (21.2)	8 (21.1)	82 (17.7)	2 (2.9)	<0.0001
Cardiovascular Death	91 (5.8)	6 (6.9)	1 (2.8)	24 (3.7)	2 (1.4)	14 (17.5)	5 (13.2)	37 (8.0)	2 (2.9)	<0.0001
non-Cardiovascular Death	75 (4.8)	3 (3.4)	2 (5.6)	15 (2.3)	4 (2.9)	4 (5.0)	3 (7.9)	44 (9.5)	0 (0.0)	
Sudden Cardiac Death	34 (2.2)	2 (2.3)	1 (2.8)	9 (1.4)	1 (0.7)	3 (3.8)	3 (7.9)	15 (3.2)	0 (0.0)	<0.0001

Values were expressed as mean ± SD, median [IQR], or counts (%) as appropriate. T2D, type 2 diabetes; LVEF, left ventricular ejection fraction; BMI, body mass index; sysBP, systolic blood pressure; diaBP, diastolic blood pressure; BNP, B-type natriuretic peptide; %GHbA1C, percentage of glycated hemoglobin A1c; HDL, high-density lipoprotein; LDL, low-density lipoprotein; NYHA, New York Heart Association Classification; ARBs, Angiotensin II receptor blocker; ACEIs, Angiotensin-Converting Enzyme Inhibitors ; β-blockers, β-adrenergic blockers; CCBs, calcium channel blockers; MI, myocardial infarction; CABG, coronary artery bypass surgery; PCI, percutaneous coronary intervention. ET-1, endothelin-1.
