# Supplementary material for: Endothelin-1 is associated with mortality that can be attenuated with high intensity statin therapy in patients with stable coronary artery disease
Source: Commun Med (Lond). 2023 Jun 22;3:87. doi: 10.1038/s43856-023-00322-9 (PMC10287654; doi:10.1038/s43856-023-00322-9)
Supplement: Supplementary file 7 — Reporting Summary [file 43856_2023_322_MOESM7_ESM.pdf]

nature portfolio

Corresponding author(s): Risto Kerkelä

Last updated by author(s): May 8, 2023

Reporting Summary

Nature Portfolio wishes to improve the reproducibility of the work that we publish. This form provides structure for consistency and transparency in reporting. For further information on Nature Portfolio policies, see our Editorial Policies and the Editorial Policy Checklist.

Statistics

For all statistical analyses, confirm that the following items are present in the figure legend, table legend, main text, or Methods section.

n/a	Confirmed	
☐	☒	The exact sample size (*n*) for each experimental group/condition, given as a discrete number and unit of measurement
☐	☒	A statement on whether measurements were taken from distinct samples or whether the same sample was measured repeatedly
☐	☒	The statistical test(s) used AND whether they are one- or two-sided<br>*Only common tests should be described solely by name; describe more complex techniques in the Methods section.*
☐	☒	A description of all covariates tested
☐	☒	A description of any assumptions or corrections, such as tests of normality and adjustment for multiple comparisons
☐	☒	A full description of the statistical parameters including central tendency (e.g. means) or other basic estimates (e.g. regression coefficient) AND variation (e.g. standard deviation) or associated estimates of uncertainty (e.g. confidence intervals)
☐	☒	For null hypothesis testing, the test statistic (e.g. *F*, *t*, *r*) with confidence intervals, effect sizes, degrees of freedom and *P* value noted<br>*Give P values as exact values whenever suitable.*
☒	☐	For Bayesian analysis, information on the choice of priors and Markov chain Monte Carlo settings
☒	☐	For hierarchical and complex designs, identification of the appropriate level for tests and full reporting of outcomes
☒	☐	Estimates of effect sizes (e.g. Cohen's *d*, Pearson's *r*), indicating how they were calculated

Our web collection on statistics for biologists contains articles on many of the points above.

Software and code

Policy information about availability of computer code

Data collection	SPSS 25 and Excel 2016
Data analysis	Kaplan-Meier survival plot were constructed by lifelines module of Python, and all statistical comparisons were performed with R version 4.0.4.

For manuscripts utilizing custom algorithms or software that are central to the research but not yet described in published literature, software must be made available to editors and reviewers. We strongly encourage code deposition in a community repository (e.g. GitHub). See the Nature Portfolio guidelines for submitting code & software for further information.

Data

Policy information about availability of data

All manuscripts must include a data availability statement. This statement should provide the following information, where applicable:

- Accession codes, unique identifiers, or web links for publicly available datasets
- A description of any restrictions on data availability
- For clinical datasets or third party data, please ensure that the statement adheres to our policy

The datasets generated and/or analyzed in the current study are available from the corresponding author upon reasonable request .

Human research participants

Policy information about studies involving human research participants and Sex and Gender in Research.

Reporting on sex and gender	Both female and male participants were included in the study. Sex is not a primary consideration when recruiting participants because the primary endpoint is all-cause of death irrespective of sex. However, male sex was considered as a candidate of risk factor based on findings from prior studies and included as a variable in multivariable analysis. The study finding was applied to both sexes. No analyses were performed on sex, given that the aim of the study was to evaluate the risk of ET-1 in all-cause of death, a protein present in both sexes.
Population characteristics	In total, there were 1946 patients in the study, and ET-1 concentration was acquired from 1945 subjects. During the follow-up (mean 7.86 ± 2.12 years), 218 subjects died with 116 due to CV death and 102 for non-CV death. 50 out of 116 CV deaths were SCDs. The overall population consisted of 31.8% (N=618) females, 42.8% (N=833) subjects with type 2 diabetes (T2D), with an average age of 67 years IQR [61, 73].
Recruitment	The inclusion criteria for the study were angiographically documented CAD with or without T2D. CAD was defined as more than one vessel with >50% stenosis detected by coronary angiography, and the diagnosis of T2D was done according to the World Health Organization standard (fasting glucose level ≥ 7.0 mmol/L or 2-hour post-load glucose level in the oral glucose tolerance test ≥ 11.1 mmol/L). The exclusion criteria included left ventricular ejection fraction (LVEF) <35%, NYHA class IV, pregnancy, life expectancy < 1 year and end-stage renal failure requiring dialysis. Blood and echocardiographic parameters were collected at the enrollment visit, the time of which was at least 3 months after the coronary angiography or the last revascularization.
Ethics oversight	The study was approved by the ethics committee of the Northern Ostrobothnia Hospital district. Written informed consent was obtained from all the participants.

Note that full information on the approval of the study protocol must also be provided in the manuscript.

Field-specific reporting

Please select the one below that is the best fit for your research. If you are not sure, read the appropriate sections before making your selection.

☒ Life sciences ☐ Behavioural & social sciences ☐ Ecological, evolutionary & environmental sciences

For a reference copy of the document with all sections, see nature.com/documents/nr-reporting-summary-flat.pdf

Life sciences study design

All studies must disclose on these points even when the disclosure is negative.

Sample size	In total, there were 1946 patients in the study, and ET-1 concentration was acquired from 1945 subjects.
Data exclusions	One participant was excluded due to no available serum sample.
Replication	The measurement of ET-1 levels from serum were performed manually and in duplicates for each sample.<br>Two statistical approaches were used to evaluate the contribution of ET-1 to the risk of endpoints: 1) a stepwise forward selection of candidate variables based on Akaike Information Criterion (forward-AIC); 2) univariable CoxPH was used to calculate variable significance, and if a result of two-sided p<0.05 was obtained, variable was included for multivariable adjustment.
Randomization	This is not relevant since this is a observational cohort study.
Blinding	This is not relevant since this is a observational cohort study.

Reporting for specific materials, systems and methods

We require information from authors about some types of materials, experimental systems and methods used in many studies. Here, indicate whether each material, system or method listed is relevant to your study. If you are not sure if a list item applies to your research, read the appropriate section before selecting a response.

Materials & experimental systems

n/a	Involved in the study
☒	☐ Antibodies
☒	☐ Eukaryotic cell lines
☒	☐ Palaeontology and archaeology
☒	☐ Animals and other organisms
☐	☒ Clinical data
☒	☐ Dual use research of concern

Methods

n/a	Involved in the study
☒	☐ ChIP-seq
☒	☐ Flow cytometry
☒	☐ MRI-based neuroimaging

Clinical data

Policy information about clinical studies

All manuscripts should comply with the ICMJE guidelines for publication of clinical research and a completed CONSORT checklist must be included with all submissions.

Clinical trial registration	URL: https://www.clinicaltrials.gov; Unique identifier: NCT01426685.
Study protocol	https://clinicaltrials.gov/ct2/show/NCT01426685
Data collection	Data were collected from during August 2007 to June 2019 and analyzed from December 2020 to April 2021.
Outcomes	The primary end point was all-cause mortality. Secondary end points were cardiovascular (CV) death, non-CV death and sudden cardiac death (SCD). SCD was defined by a witnessed death within one hour of the onset of symptoms, or unwitnessed death where the patients was last seen alive and stable within 24-hours before found deceased.
